# Phonemic Training Modulates Early Speech Processing in Pre-reading Children

**DOI:** 10.3389/fpsyg.2021.643147

**Published:** 2021-06-01

**Authors:** Anne Bauch, Claudia K. Friedrich, Ulrike Schild

**Affiliations:** Developmental Psychology, Department of Psychology, University of Tuebingen, Tuebingen, Germany

**Keywords:** phonemic awareness training, precursors of reading acquisition, event-related potentials, speech processing, children

## Abstract

Phonemic awareness and rudimentary grapheme knowledge concurrently develop in pre-school age. In a training study, we tried to disentangle the role of both precursor functions of reading for spoken word recognition. Two groups of children exercised with phonemic materials, but only one of both groups learnt corresponding letters to trained phonemes. A control group exercised finger-number associations (non-linguistic training). After the training, we tested how sensitive children were to prime-target variation in word onset priming. A group of young adults took part in the same experiment to provide data from experienced readers. While decision latencies to the targets suggested fine-grained spoken word processing in all groups, event-related potentials (ERPs) indicated that both phonemic training groups processed phonemic variation in more detail than the non-linguistic training group and young adults at early stages of speech processing. Our results indicate temporal plasticity of implicit speech processing in pre-school age as a function of explicit phonemic training.

## Introduction

Previous research has provided evidence for an intimate relationship between speech processing and literacy. Experienced readers who command an alphabetic writing system show spelling biases in some purely auditory tasks requiring specific linguistic decisions. Spelling biases appeared in lexical decisions, requiring participants to decide whether a spoken string is a word or not. Experienced readers recognized spoken words with rhymes that can have several spellings (e.g., “flow–though”) slower than they recognized spoken words that have consistently spelled rhymes (e.g., “house–mouse”; Ventura et al., 2004; Ziegler et al., 2004, 2008; Pattamadilok et al., 2007, 2014; Perre et al., 2011). Spelling biases also appeared in phoneme detection, requiring participants to indicate the presence of a specific speech sound in a spoken word or in a non-sense word. In auditory tasks, experienced readers detected phonemes that can have several spellings (e.g., /f/ in “phone” or “foam”) slower than they detected consistently spelled phonemes (e.g., /m/ in “moldy” or “monkey”; Cutler et al., 2010). Such spelling biases in tasks that involve purely spoken material have inspired a lively discussion on how the relationship between reading and speech processing is natured. In sum, this research implies that orthographic knowledge can slow down auditory word recognition in skilled readers, which might be disadvantageous for speech comprehension. Inversely, the relationship between speech processing and reading might also have an advantageous side: Building representations of newly acquired graphemes might feedback to already existing phoneme representations thereby strengthen phoneme representations which might help to discriminate phonemes more reliable. Here, we aim to investigate the latter and approach this discussion from a developmental perspective by providing data from a training study on precursor functions of reading acquisition.

According to interactive accounts of spoken and written word recognition (e.g., Grainger and Ferrand, 1996; Ziegler and Ferrand, 1998; Ziegler et al., 2003), newly acquired grapheme knowledge should link to phonological representations that are relevant for lexical access. According to this view, grapheme representations might automatically be co-activated with phonological representations of single phonemes, when listeners attend to speech related auditory input. It is assumed that activated grapheme representations then interfere directly with lexical access, e.g., by sending additional activation to respective phonological representations. In the following article, we will refer to this approach, which highlights implicit, automatic or obligatory grapheme-phoneme links, as the *implicit orthographic-phonological account*. Another, different approach stresses that reading and reading acquisition might fundamentally restructure phonological representations through intensive phonological processing by a listener's increasing awareness for the structure of the language (e.g., Harm and Seidenberg, 2004; Taft, 2006; Pattamadilok et al., 2010; Dehaene et al., 2015). According to this second account, spelling biases might reflect facilitated phonological processing of consistently spelled words rather than arise out of an obligatory activation of orthographic knowledge in spoken word recognition. As this account also focusses on rather automatic processes of word recognition, we will refer to it as *implicit phonological account*. It should be noted that although in research there is a lively discussion about whether reading affects word recognition via the one or the other approach, both accounts are not necessarily exclusive. As in, reading acquisition might trigger a restructuring of the phonological system, leading to more distinctive phonological representations. Additionally, grapheme representations might simultaneously be co-activated during word recognition, and feed activation to respective phonological representations. In contrast to both former accounts, a third approach restricts effects of reading and reading acquisition to explicit metalinguistic aspects that are not necessary for lexical access (e.g., Cutler et al., 2010; Cutler and Davis, 2012; Mitterer and Reinisch, 2015). According to this third account (in the following referred to as *explicit phonological account*), only a metalinguistic level of thinking about speech profits from reading acquisition whereas obligatory or implicit aspects of speech processing are not tapped by reading experience.

Coincident development of grapheme knowledge and phonological awareness in middle childhood makes it difficult to dissociate between the modulating role that either phonemic awareness (the awareness for single sounds) and/or grapheme knowledge exert on speech recognition. When children become aware of phonological units like rhymes, syllables or phonemes, they typically also learn that there are letters (or graphemes) which correspond to the speech sounds (e.g., Torgesen et al., 1994). Thereafter, when they start formal reading instruction, children explicitly learn to associate sounds with graphemes in close correspondence with explicit phonemic awareness. Children learn, for example, that the words “bad” and “bag” comprise three phonemes which relate to three graphemes and that both words differ only in the third phoneme and grapheme, respectively. It is therefore not surprising that explicit phonological awareness and orthographic knowledge are reciprocally related (Perfetti et al., 1987; Cataldo and Ellis, 1988; Wagner et al., 1994). Children's sensitivity for rhymes, syllables and especially phonemes predicts later reading skills (Wagner and Torgesen, 1987; Naslund and Schneider, 1996; Ehri et al., 2001; Castles and Coltheart, 2004; Melby-Lervåg et al., 2012) and their orthographic knowledge interferes with performance on explicit metalinguistic tasks (Ehri and Wilce, 1980; Tunmer and Nesdale, 1985; Treiman and Cassar, 1997; Castles et al., 2003). Thus, at least in alphabetic writing systems, phonological awareness and emerging literacy mutually complement each other [see Cheung et al. (2001) for reduced explicit phonological awareness in readers of non-alphabetic logographic scripts].

A previous study obtained indirect evidence for plasticity of speech processing in relation to early reading abilities (Schild et al., 2011). In a cross-sectional design, the study investigated pre-reading and reading pre-schoolers as well as reading pupils by means of word onset priming. Participants listened to spoken prime-target combinations in three different conditions. In an Identity condition, spoken word onset primes completely overlapped with consecutively presented spoken targets (e.g., “mon–monster”). In two other conditions, primes either varied in the initial place of articulation from their targets (Variation condition, e.g., “non–monster”), or primes and targets differed completely (Control condition, e.g., “dak–monster”). Participants made lexical decisions to the targets. Reading and pre-reading children responded faster to targets in the Identity condition compared to the Control condition, but only readers responded faster to the Identity condition compared to the Variation condition. This suggested that, compared to pre-readers, readers were more sensitive to subtle phonological variation. However, lexical decision latencies in word onset priming appear relatively late and reflect several aspects of the complex recognition process including selection of appropriate word candidates among those matching or partially mismatching the input (e.g., Friedrich et al., 2013; Schild and Friedrich, 2018). Therefore, group differences in decision latencies as a function of prime-target overlap can also be explained with the *explicit phonological account* (e.g., Cutler et al., 2010; Cutler and Davis, 2012; Mitterer and Reinisch, 2015). However, response latencies do not allow to draw conclusions on whether also early implicit aspects of speech processing are tapped by literacy acquisition.

In this previous study (Schild et al., 2011), event-related potentials (ERP) preceding the lexical decision responses revealed plasticity of implicit speech processing. Between 300 and 400 ms after target word onset, anterior ERP amplitudes for reading children differed between the Identity condition and the Variation condition. For pre-readers, ERP did not differ between both conditions. The authors related ERP differences between the Identity and the Variation condition in readers to the left-anterior P350 emerging between 300 and 400 ms after target word onset in adults (Friedrich, 2005; Friedrich et al., 2008, 2009, 2013; Bien et al., 2014; Schild et al., 2014b; Kóbor et al., 2018; Schild and Friedrich, 2018). For adults, P350 effects in word onset priming reflected fine-grained mapping between the input and lexical representations (e.g., Friedrich et al., 2009; Schild et al., 2012). Thus, it was suggested that in reading children, implicit lexical access either was modulated by the newly acquired letter knowledge (similar to the *implicit orthographic-phonological account*) or by more fine grained lexical representations (similar to the *implicit phonological account*, Schild et al., 2011). For syllable stress, which is a phonological component not encoded in the German writing system, neither lexical decision latencies nor ERP recorded in word onset priming indicated processing differences between reading and pre-reading children (Schild et al., 2014b). This implied that group differences found for the processing of subtle phonological variation (Schild et al., 2011) are restricted to phonemes as most relevant units for reading an alphabetic language.

The present study aimed to elucidate more directly whether phonemic awareness alone or additional grapheme knowledge is associated with more detailed implicit processing of speech observed in readers. To our knowledge, this is the first study to implement a specific training in pre-reading children in order to unravel the relationship between grapheme knowledge, phonemic awareness and speech processing. We carried out daily 10-min game-like interventions for 10 weeks with pre-reading kindergarteners. Across three groups of children, we realized three different interventions. (i) One group received a daily training on phonemic awareness (phonemic group), (ii) a second group received a combined phonemic awareness and grapheme knowledge training (phonemic-orthographic group), and (iii) a third group received a training not focusing on language development, but on finger-number associations (non-linguistic control group). After the training, we recorded ERP and lexical decision latencies in an auditory word onset priming experiment with all participants in individual test sessions [see Schild et al. (2011)]. We aimed to investigate the following research questions:

### Can Previous Findings on Tolerance for Feature Mismatch Between the Prime Syllable and the Target Word in Pre-school Children be Generalized From Place of Articulation to Voicing Feature?

To make results comparable between the former and the present study we applied the same experimental word onset priming paradigm. We tested whether preliterate children's tolerance to phonemic variation can be generalized to manipulations in other phonemic features than place of articulation (Schild et al., 2011). If pre-readers indeed depict a more general tolerance to subtle phonemic variation, we should be able to replicate the previous findings in pre-reading children with voicing variation. Results of the control group receiving a non-linguistic training of precursor functions of mathematical skills will be indicative for this question. In accordance with previously obtained results for untrained pre-readers (Schild et al., 2011), we expected that control children should not show fine-grained priming effects (neither in the ERPs nor in the lexical decision latencies, i.e., we expect no difference between the Identity condition (e.g., “ki–kino”) and the Variation condition (e.g., “gi–kino”), but both conditions should differ from the Control condition (e.g., “ba–kino”). Finally, a group of young adults provided data for experienced readers. Here, we expected the same graded activation patterns for voicing variation (i.e., differences between all conditions) as previously found in adult participants for place of articulation variation for both lexical decision latencies and P350 amplitudes (e.g., Friedrich et al., 2009; Schild and Friedrich, 2018).

### Does a Training of Phonemic Awareness Alone or Combined Training of Phonemic Awareness and Letter Knowledge Lead to More Detailed Speech Processing in Preliterate Children?

We considered comparison of both phonemic training groups to be informative about the contribution of phonemic awareness alone or in combination with grapheme knowledge to refined implicit speech processing in children. Both phonemic training groups received training to detect phonemic variations. The daily interventions focused on two specific German consonants differing in voicing (/g/ and /k/). In a word onset priming experiment after the training, these two trained phonemes were compared to two untrained phonemes differing in voicing (/b/ and /p/). We hoped the comparison between trained and untrained phoneme pairs as to be informative about how the training of phonemic awareness and letter knowledge might affect the processing of phonological mismatch. There are very few training studies that have explicitly investigated the extent to which a training of phonemic awareness of specific phonemes generalizes to sensitivity also for other phonemes. Evidence for a generalization also to untrained phonemes can be found, for example, in the study of Byrne and Fielding-Barnsley (1991), in which children's phoneme identification skills did not only increase for trained phonemes, but also for phonemes that were not practiced in the training itself. Based on the fact that the training exercises overarching metalinguistic skills, we assume that phonemic sensitization should generalize and not be limited to the trained set of phonemes. If phonemic training also generalizes to untrained phonemes, this should be reflected in graded priming-effects in response latencies and P350-amplitudes comparable to those found for reading children in the former study by Schild et al. (2011), in both phonemic training groups. In particular, ERP differences would be evidence for *implicit phonological accounts* focusing on phonological representations rather than grapheme representations (e.g., Harm and Seidenberg, 2004; Taft, 2006; Pattamadilok et al., 2010; Dehaene et al., 2015). If grapheme knowledge boosts refinement of implicit speech processing additionally, the combined phonemic-orthographic training group should show most pronounced P350 priming effects in speech processing compared to both other groups, especially for the trained phonemes. This would be evidence for an intimate relationship between speech sound representations and grapheme representations as assumed by the *implicit orthographic-phonological account* (e.g., Grainger and Ferrand, 1996; Ziegler and Ferrand, 1998; Ziegler et al., 2003). As graphemes map relatively specifically to certain phonemes, in this case additionally activity from the graphemes should only spread to trained phonemes, but not untrained ones. If group differences are restricted to response latencies, this would be evidence in support of *explicit phonological accounts* (e.g., Cutler et al., 2010; Cutler and Davis, 2012; Mitterer and Reinisch, 2015).

## Methods

### Training Study

Participating children took part in one of the two language trainings or in the non-phonemic control training. Children of the language groups were either part of a phonemic-orthographic training group (PHORT) or a phonemic-only training group (PHON). In both experimental groups, children received a phonemic-orientated training that focused on the awareness of initial sounds and phoneme synthesis and analysis. In both of these groups, the training primarily aimed to sensitize the children for the contrast between the two phonemes /g/ and /k/. In the phonemic-orthographic group, the children additionally learned the corresponding letters G and K. In contrast, participants of the non-linguistic training (CONTROL) were trained with numerical material only, and exercised with short games aiming to train finger-number-associations (including finger gnosis tasks, ordinal and cardinal finger-number associations and number relations). Here, we only focus on the conduction and the results of the two linguistic trainings. For detailed information about the numerical control training, the content of the control training and and its results, see Schild et al. (2020).

The trainings were randomly assigned to and conducted in local kindergartens in the city of Tuebingen, Germany. To prevent distraction caused by the daily kindergarten routine, the trainings were all held in quiet, separate rooms in the kindergartens. All participants were in their final year of kindergarten, i.e., they were between 5 and 6 years of age. Children within the same kindergarten received the same training. We allowed bilingual children to take part in the training itself to maintain the integrity of the pre-school groups but they were excluded from further testing. All three trainings consisted of 50 daily sessions and ran for ~10 weeks from February/March to May/June in 2015 and 2016. Kindergartens participating in both years switched training form in the second year to control for possible environmental effects. Each daily session lasted for 10–15 min and was led by instructed collegiate and doctoral members (Bachelor students and Ph.D. candidates) of the Department of Psychology, Eberhard-Karls-University Tuebingen, Germany. In each kindergarten, the training was led by two trainers at a time. A manual with specific instructions for the conduction of the short games was created for each training form to ensure standardization of the trainings. Before the interventions started, each trainer was briefed about how to conduct the trainings.

Before and after the training period we tested each child in two individual sessions (each lasting for about 30–40 min) in order to obtain measurements of language, arithmetical and general cognitive abilities. These tests were conducted in local rooms of the kindergartens. Finally, each child participated in a reaction time experiment with EEG recording at our laboratory. This session lasted for 2 h on average, with the EEG experiment lasting for about 30 to 40 min.

### Participants

Initially 102 children participated in the training study. Children and parents received written information about the study and signed a letter of consent. Parents provided demographic and developmental information about their child in a questionnaire. The children received a present after each of the individual sessions. Originally, *n* = 30 children received a combined phonemic-orthographic training, *n* = 37 children received a phonemic-only training and *n* = 35 received the control training. After applying the exclusion criteria as listed below, we had to exclude *n* = 6 data sets provided by children from the combined phonemic-orthographic group, *n* = 15 data sets obtained by children from the phonemic-only training and *n* = 14 data sets that originated from children from the control group. In detail, we had to exclude 21 datasets, because respective EEG recordings were either too noisy or failed to contribute enough segments for ERP analysis (we pre-defined a minimum of 15 segments). Another twelve datasets were lost because children refused to wear the cap for the EEG recording (*n* = 6), or because they were unavailable for EEG testing altogether (*n* = 6). Five children were identified with early reading skills via the parental questionnaire, by asking the children about their reading and writing skills and pre-testing with the reading test “Ein Leseverständnistest für Erst- bis Sechstklässler” (ELFE 1-6; Lenhard and Schneider, 2006) and were also excluded from data analysis. We considered them as children with early reading skills if they were able to read aloud single unknown words at the word comprehension subtest of the ELFE 1-6. Note that the higher number of reading pre-schoolers in the study by Schild et al. (2011) reflects the former's recruiting strategy. In their study, they actively searched for reading pre-schoolers by respective announcements in local newspapers. Yet, given our research questions, we were not interested in reading pre-schoolers in the present training study. Finally, another three children were excluded due to increased error rates in the behavioral experiment (cut-off rate for missing words > 20%, *n* = 3; for incorrect responses to pseudowords > 80%, *n* = 1). The final sample included datasets of 67 children.

All remaining children grew up in a monolingual German-speaking environment. Table 1 sums up more detailed demographic information. None of the children was able to properly read and write. However, nearly all children were able to write their first names and/or the names of their parents/siblings. There were no reports of neurological or hearing problems for any of the children. All participants had normal or corrected-to-normal eyesight. There were no significant differences in general cognitive abilities between the three groups as measured by the *Matrices* subtest of the “Culture Fair Intelligence Test 1—Revision” (CFT 1-R; Weiß and Osterland, 2013). Handedness for all participants was assessed via the “Edinburgh Handedness Inventory” (EHI; Oldfield, 1971). The Ethics Committee of the German Psychological Association (Ethikkommission der Deutschen Gesellschaft für Psychologie) approved of this study.

**Table 1:** Demographic information on distribution of sex across the groups, mean (*M*) and standard deviations (*SD*) for age in months at post-testing, days of attendance in the training and mean results of the standardized tests on general cognitive abilities (max = 15, CFT 1-R, *Matrices* subtest, Weiß and Osterland, 2013) and handedness *Lateralization Quotient* (LQ; Oldfield, 1971) of the phonemic-only (PHON), phonemic-orthographic (PHORT) and non-linguistic control (CONTROL) group.

	**Group**			**Group diff**.	
**Variable**	**PHON**	**PHORT**	**CONTROL**	***F***	***p***
*N*	24	22	21		
Sex female/male	10/14	10/12	10/11		
Age in months *M* (*SD*)	74.10 (4.92)	73.81 (3.84)	73.80 (5.01)	0.55	0.57
Attendance in days *M* (*SD*)	38.97 (8.40)	42.02 (5.74)	40.26 (7.20)	1.02	0.36
CFT1-R *M* (*SD*)	7.50 (3.38)	7.18 (3.49)	5.71 (3.83)	1.61	0.20
LQ *M* (*SD*)	45.95 (66.02)	55.57 (52.74)	72.25 (45.23)	1.26	0.29

*There were no significant differences between groups (Group diff.) in age, CFT1-R, attendance and LQ measurements*.

We additionally recruited 18 young adult participants (nine females) who were all students of the University of Tuebingen (mean age = 23 years). All adult participants were right-handed and native speakers of German. None of them reported neurological or hearing problems. All of them had normal or corrected-to-normal eyesight. Adult participants received oral and written information about the study and signed consent to participate in the experiment. They were compensated for their efforts with either eight Euros per hour or course credits.

### Training Material

For the language groups, procedure and games were adapted from Küspert and Schneider (2008) and Plume and Schneider (2004). Both language trainings contained the same 15 phonemic games (see Supplementary Table 1). While the phonemic-only group received an auditory phonemic training only, the phonemic-orthographic group additionally received training in phoneme-grapheme-mappings for the trained phonemes. The games varied daily and increased in their level of difficulty during the course of the training program. The training was based on the German voiced and voiceless consonants /g/ and /k/. We supplemented the training material with the vowels /a/, /e/, /i/, and /o/ to provide an easy access and to facilitate the familiarization with the exercises. Two sets of in total 57 picture cards served as the main material for the games. For the phonemic-orthographic group, we complemented the material with respective capital letter cards (A, E, I, O, G, and K).

We focused on exercises that targeted the training of phonemes. For instance, in the *Picture Card Game* children picked one of the cards and then named the initial phoneme of the illustrated object (and assigned them to the corresponding letter card in the phonemic-orthographic group, respectively). While the first half of the training concentrated on phoneme onset detection games only, the second half of the training also included exercises on phoneme synthesis and analysis. For example, here, children were asked to segment words into their single phonemes (e.g., the *Sound Ball* game, phonemes of a word were given spelled separately and children were asked to name the word as a whole; “*Which word do I mean? K-I-W-I*”). Target words from the behavioral experiment were not included as training material to avoid familiarity effects. Length and organization of the training for the non-linguistic control training was comparable to that of the two language groups. For more information about the training material of the control training, see Schild et al. (2020).

### Pre- and Post-tests

Phonological awareness, phonemic awareness and letter knowledge were tested before and after the ten-week training period. Handedness and general cognitive abilities were assessed once after the training period.

#### Phonological Awareness

Phonological awareness was measured with the “Test zur Erfassung der phonologischen Bewusstheit und Benennungsgeschwindigkeit” (TEPHOBE; Mayer, 2011). The TEPHOBE contains the four subtests *Synthesis of Onset and Rhyme, Phoneme Synthesis, Rhyming*, and *Categorization of Initial Sounds* whereby all except the rhyming subtest could in general be affected by the phonemic training. Each subtest consisted of 7 items, resulting in a total score of 28 (cronbach's α for the subtests and total score ranging between 0.71 and 0.78, as according to the manual). A score of 1 was given for each correct answer. As a control two additional subtests capture rapid automatized naming (*Naming of Colors* and *Naming of Objects*). Underlying skills of these control tests were not subject of the phonemic training. There was no child that performed below average on this test.

#### Phonemic Awareness

Phonemic awareness was tested with an additional test (*Spy Game*) which we adapted from Castles et al. (2011). This card-based game consisted of 13 different objects all starting with a different phoneme, e.g., “Gitarre” (*guitar*), “Birne” (*pear*), “Apfel” (*apple*). At first, we asked all participants to name all of the objects presented on the cards to check whether they recognized the objects. If a child used a different name for one of the objects, the instructor corrected with the intended name. Following this, children were asked to identify the object that start with a specific sound (e.g., “I can see an object starting with /g/, can you name it?”). A score of 1 was given for each correct answer. This set included both the trained consonants /g/ and /k/ and the untrained consonants /b/ and /p/, which were part of the subsequent behavioral experiment. The remaining objects started with the vowels (/a/, /e/, /o/, and /u/) and the additional consonants (/d/, /f/, /m/, /n/, /t/), which were unrelated to the subsequent behavioral experiment.

#### Letter Knowledge

The children were asked to name 15 letters in upper case and lower case. Like the *Spy Game, Letter Knowledge* tested the knowledge of the trained and untrained consonants as well as vowels and additional untrained consonants (G, K, B, P, A, E, I, U, O, D, T, S, W, H, R). A score of 1 was given for each correct answer.

#### General Cognitive Abilities and Handedness

Children performed the two subtests *Matrices* and *Continuing Rows* of the “Culture Fair Intelligence Test–Revision” (CFT 1-R, Weiß and Osterland, 2013) for an estimation of their cognitive abilities. However, since instructors had repeatedly reported that children had difficulties with the subtest *Continuing Rows*, only results from the *Matrices* subtest entered analyses. The subtest consisted of 15 items, with a score of 1 given for each correct answer (cronbach's α = 0.89, according to the manual). There was no child that performed below average on this test. The handedness of each participant was measured with the “Edinburgh Handedness Inventory” (EHI, Oldfield, 1971). Note that we omitted the item “Striking A Match.”

#### Reading Abilities

Before pre-testing, we screened all parental questionnaires to detect children with advanced reading skills. Parents were asked to rate the reading and writing skills of their child. Additionally, at the pre-test and the post-test, the children themselves were asked about whether and what they were able to read and write, to write down their name and any other word they could write. If the information given by the parents and the child indicated that the child was able to write any word other than their name or the name of another person, the child additionally completed the reading test “Ein Leseverständnistest für Erst- bis Sechstklässler” (ELFE 1-6; Lenhard and Schneider, 2006) in estimation of the child's level of reading proficiency.

As the non-linguistic control training served as an experimental group for a research question of its own, we also measured children's levels of finger-gnosis and basic arithmetic skills (Schild et al., 2020).

### Experimental Stimuli and Procedure

Seventy-four monomorphemic disyllabic German nouns served as targets (see Supplementary Table 2). All of them were stressed on the first syllable. Half of the word started with one of the trained phonemes (either /g/ or /k/); the other half of the words started with one of the untrained phonemes (either /b/ or /p/). Furthermore, we added 74 pseudowords for the lexical decision task. Here, we extracted the second syllable of each target word and exchanged them with the second syllable of another target word. For example, the second syllable of “Bre*zel*” (*pretzel*) was inserted as the second syllable in “Ket*te*” (*chain*) and vice versa, resulting in the two pseudowords “Bre*te*” and “Ket*zel*.”

Primes were created from the first syllable of each target word. The prime-target combination varied across three experimental conditions. In the Identity condition, prime and target completely matched (e.g., “ki–kino”). In the Variation condition, the prime varied from its assigned target in the voicing of its initial sound (e.g., “gi–kino”). In the Control condition, the prime and the target were unrelated insofar as their first syllables contained different phonemes and, additionally, the first phoneme differed in the place of articulation as well as in the voicing to maximize differences between prime and target (e.g., “ba–kino”). A pseudoword appeared instead of a target in 33% of the trials. Primes and pseudowords were combined according to the different conditions in the same way as the primes and targets. Every target appeared once in every condition, every pseudoword appeared only once in total. The stimulus material was recorded by a male and a female professional native speaker of German. The primes were taken from words spoken by the male speaker and the targets and pseudowords were taken from the female speaker to prevent mere acoustical priming effects. Both speakers were unaware of the purpose of the study.

Children and adults completed a unimodal auditory word-fragment priming experiment with EEG recording. In total, 296 trials (222 targets and 74 pseudowords) were presented, which appeared in twelve blocks. In eight blocks, participants listened to 25 trials and in four blocks to 24 trials. Targets were not repeated within a block. Trials were randomized within each block. Across participants, the sequence of the blocks was balanced. We introduced the experiment as a “word-catching-game.” Participants were instructed to press the space bar as fast and as correctly as possible whenever they heard a real word and to refrain from responding whenever they heard a pseudoword. Each trial started with the presentation of a fixation picture (1 × 1 cm, a smiley) in the middle of the screen. After 500 ms the auditory prime was presented. The auditory target or a pseudoword followed 200 ms after offset of the prime to create a comparable and adequate baseline period for the ERP. Brief visual feedback (3 × 7 cm) was provided whenever the child responded correctly to a target (a smiley flying into a basket), or whenever the child incorrectly pressed the space bar for a pseudoword (a little ghost appeared in the middle of the screen). The next trial started 1.5 s after the offset of the feedback. No feedback was given whenever the child missed a target. In this case, the next trial started 3.5 s after the onset of the target. The total duration of the priming experiment itself was ~20 min without breaks, and about 30 to 45 min with breaks, depending on the child. After each block a short break was provided.

To minimize movement artifacts in the EEG signal, we seated participants as comfortably and relaxed as possible on a solid chair in ~50 cm distance to the screen. They were instructed to fixate the middle of the screen. The stimuli were presented via loudspeakers placed on both sides of the screen. The intensity of the sound stimuli was kept constant for all children at 40% volume. The experiment was conducted in an electrically shielded and sound-attenuated room. Half of the participants used the index finger of their right hand, and the other half of the participants used the index finger of their left hand to press the space bar.

### Electrophysiological Recording

We used 46 active Ag/AgCl electrodes (Brain Products) attached into an elastic cap (Electro Cap International, Inc.) for the continuous EEG recording according to the international 10–20 system (bandpass filter 0.01–100 Hz, BrainAmp Standard, Brain Products, Gilching, Germany). The reference and the ground electrode were placed on the tip of the nose and in the electrode cap at position AF3, respectively. Two additional electrodes were placed below each eye. Two eye-calibration blocks were presented before and after the experiment. EEG data was processed with the Brain Electrical Source Analysis Software (BESA, MEGIS Software GmbH, Version 5.3). We applied the surrogate Multiple Source Eye Correction (Berg and Scherg, 1994) implemented in BESA for eye-movement artifact correction. For offline analysis, the signal was re-referenced to an average reference. All remaining artifacts (e.g., head or facial movements) were rejected manually and by visual inspection. Individual noisy channels were linearly interpolated for all trials (*M* = 3.65, *SD* = 1.44, *Range* = 0–6 for children, *M* = 1.27, *SD* = 1.36, *Range* = 0–4 for adults). All recordings were filtered offline with a 0.3 Hz high-pass filter. ERP were computed only for correctly identified targets, starting from the beginning of the speech signal until 700 ms post-stimulus onset, with a 200 ms pre-stimulus baseline.

### Data Analysis

#### Explicit Tests

A repeated measures ANOVA with the within-factor *Time* (pre-test vs. post-test) and the between-factor *Group* (PHORT vs. PHON vs. CONTROL) was applied. If assumptions of normal distribution or variance homoscedasticity were violated, non-parametric Welch-Test were applied.

#### Reaction Times and Errors

Reaction times (RT) shorter than 200 ms and longer than 2,000 ms were removed from analysis. A repeated measures ANOVA with the within-factors *Condition* (Identity vs. Variation vs. Control) and *Phoneme* [trained phonemes (g/k) vs. untrained phonemes (b/p)] and the between-factor *Group* (PHORT vs. PHON vs. CONTROL vs. ADULT) was applied. The same procedure was used for the analysis of errors in word trials (omissions). The RT result section will report significant main effects of the factors *Condition* and *Group* and interaction effects including these factors.

#### Event-Related Potentials

Four regions of interest (ROI, anterior-left: F9, F7, F3, FT9, FT7, FC5, FC1, T7, C5; posterior-left: C3, TP9, TP7, CP5, CP1, O9, P3, PO9, O1; anterior-right: F10, F8, F4, FT10, FT8, FC6, FC2, T8, C6; posterior-right: C4, TP10, TP8, CP6, CP2, P8, P4, PO10, O2) were identified prior to analyses. ERP amplitudes were computed with the same ANOVA as the reaction times, with the additional factors *Region* (anterior vs. posterior) and *Hemisphere* (left vs. right). In order to make the present analysis comparable to the results of the study by Schild et al. (2011), we adapted the same time windows in the present study. This resulted in a first time window ranging from 100 to 300 ms and a second time window from 300 to 400 ms. Both time windows preceded the behavioral responses. The ERP result section will report significant interactions of *Condition* with *Group, Phoneme, Hemisphere* and/or *Region*. In case of significant interactions, follow-up ANOVAs and *t*-tests were computed. All reported *t*-test results were subject to Holm-Bonferroni correction.

Note that in all group comparisons in the section for reaction times, error analysis and ERP analysis, we included the young adults as a group in the ANOVA. The same analysis were also calculated with only the three children groups. Group comparison results with and without the adult control group differed only marginally from each other and made no differences in significances and effect sizes for the ERP results. In the analysis for reaction times, there was an additional main effect for Group when the adult group were inserted in the analysis (see section Results). Detailed results of the analysis without the adult group can be found in Supplementary Table 3 (reaction times and error analysis) and Supplementary Table 4 (ERP analysis) in the Supplementary Material.

## Results

### Explicit Tests

Table 2 depicts mean scores for the pre- and post-tests. At pre-test, there were no significant group differences in *phonological awareness, phonemic awareness* and *letter knowledge*, all *Fs*_(2, 64)_ ≤ 2.92, n.s.

**Table 2:** Mean values (*M*) and standard deviations (*SD*) for all specific tests for pre-test and post-test point of measurement, separated for each group (PHON, phonemic only group; PHORT, phonemic-orthographic group; CONTROL, non-linguistic control group).

	**Group**						**Pre-test group diff**.	
	**PHON**		**PHORT**		**CONTROL**			
**Test**	**Pre-test *M* (*SD*)**	**Post-test *M* (*SD*)**	**Pre-test *M* (*SD*)**	**Post-test *M* (*SD*)**	**Pre-test *M* (*SD*)**	**Post-test *M* (*SD*)**	***F***	***p***
**SPY GAME**								
*Total score* Max = 13	7.58 (4.28)	9.29 (2.88)	6.27 (4.33)	8.50 (3.55)	4.95 (4.12)	6.47 (3.78)	2.11	0.12
**LETTER KNOWLEDGE**								
*Total score capital letters* Max = 15	8.79 (4.28)	10.91 (3.93)	9.04 (4.29)	10.86 (4.14)	6.23 (4.28)	7.80 (5.16)	2.92	0.08
*Total score small letters* Max = 15	5.33 (4.32)	7.66 (3.93)	6.22 (4.17)	7.31 (4.16)	3.61 (3.04)	5.00 (4.25)	2.71	0.08
**TEPHOBE**								
*Total score* Max = 28	19.25 (5.29)	22.62 (3.86)	18.90 (4.32)	20.81 (3.87)	17.76 (4.36)	18.47 (4.70)	0.61	0.54
*Onset and rime* Max = 7	5.87 (1.29)	6.54 (0.93)	5.50 (1.26)	6.40 (0.90)	6.00 (1.44)	5.95 (1.20)	0.83	0.44
*Phoneme synthesis* Max = 7	5.33 (1.40)	6.58 (0.97)	4.95 (1.98)	5.77 (1.41)	4.71 (1.67)	5.38 (1.32)	0.76	0.47
*Rhyming* Max = 7	4.66 (1.90)	5.37 (1.55)	5.40 (1.68)	5.18 (1.50)	4.47 (2.15)	4.80 (2.08)	1.44	0.24
*Initial sound* Max = 7	3.37 (1.78)	4.12 (2.19)	3.04 (2.21)	3.45 (2.57)	2.57 (1.69)	2.42 (1.96)	0.99	0.37
*Speed naming objects* (items/sec)	0.66 (0.16)	0.66 (0.18)	0.70 (0.19)	0.70 (0.15)	0.61 (0.13)	0.65 (0.15)	1.56	0.21
*Speed naming colors* (items/sec)	0.61 (0.19)	0.65 (0.24)	0.65 (0.22)	0.68 (0.22)	0.57 (0.12)	0.60 (0.16)	0.97	0.38

*Test statistics of pre-test group comparisons (Pre-test group diff). There were no significant differences in the explicit tests between the groups prior to the training*.

#### Phonological Awareness

We found a significant interaction of *Group* × *Time* in the phonological awareness test (TEPHOBE, total score), *F*_(2, 64)_ = 2.97, *p* = 0.05, η*p*^2^ = 0.08. Both phonemic groups scored higher in the total score after the training, PHON: *t*_(23)_ ≥ 4.27, *p* ≤ 0.0002, *d* = 0.82; PHORT: *t*_(21)_ ≥ 2.14, *p* ≤ 0.04, *d* = 0.41. This was not the case for the CONTROL group, *t*_(20)_ ≤ 1.17, n.s. In the subtests of the phonological awareness test we found a significant main effect of *Time* in the subtests *Onset and Rhyme* and *Phoneme Synthesis*. Children of all three groups scored higher in these subtests after the training, both *ts*_(1, 64)_ ≥ 2.97, *p* ≤ 0.004, *d* ≥ 0.44. There were no main effects of *Time* [all *ts*_(1, 64)_ ≤ 1.54, n.s.] in any of the other subtests. Furthermore, there were no main effects of *Group* [all *ts*_(2, 64)_ ≤ 2.88, n.s.] nor interaction effects of *Group* × *Time* [all *ts*_(2, 64)_ ≤ 1.62, n.s.] for any of the subtests.

#### Phonemic Awareness

The ANOVA revealed a significant main effect of *Time* for the test of phonemic awareness (Spy Game), *F*_(1, 64)_ = 20.82, *p* < 0.001, η*p*^2^ = 0.25. Children of all training groups recognized more phonemes after the training than before the training, *t*_(66)_ = 4.62, *p* < 0.001, *d* = 0.52. Additionally we found a main effect of *Group* in the phonemic awareness test, *F*_(2, 64)_ = 3.40, *p* = 0.040, η*p*^2^ = 0.10. Children from the phonemic-only training group scored higher in the phonemic awareness test than children from the control group with non-linguistic training, *t*_(41.27)_ = 2.66, *p* = 0.01. We neither found significant performance differences in the *t*-test comparing the combined training group and the control group with non-linguistic training [*t*_(40.49)_ = 1.51, n.s.], nor in the *t*-test comparing the combined training group and the phonemic-only group, *t*_(42.12)_ = 1.01, n.s.

#### Letter Knowledge

We found main effects of *Time* for all scores of letter knowledge (capital and small letters), both *Fs*_(1, 64)_ ≥ 25.03, *p* ≤ 0.001, η*p*^2^ ≥ 0.28. All children knew more letters after the training compared to pre-test point of measurement, both *ts*_(66)_ ≥ 10.59, *p* ≤ 0.001, *d* ≥ 0.63. Furthermore, a main effect of *Group* was significant in the *letter knowledge* of capital letters, *F*_(2, 44)_ ≥ 3.44, *p* ≤ 0.038, η*p*^2^ = 0.10. Compared to the non-linguistic control training group, the phonemic-orthographic group knew more capital letters, *t*_(40.77)_ = 2.51, *p* = 0.01. There was no difference between the control and phonemic-only group [*t*_(42.82)_ = 1.49, n.s.], nor between both phonemic training groups, *t*_(43.99)_ = 0.97, n.s.

### Reaction Time and Error Analysis

**Figure 1:**
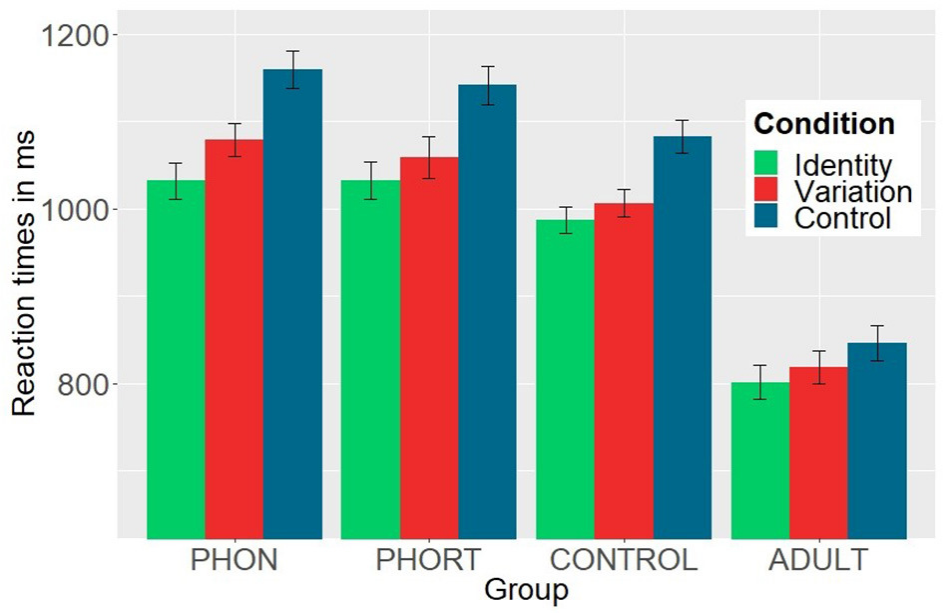
Mean reaction times and standard deviations (as indicated by error bars) of the three conditions (Identity, Variation, and Control) for each training group (PHON, phonemic only group; PHORT, phonemic-orthographic group; CONTROL, non-linguistic control group) and adult control group (ADULT).

Figure 1 presents mean reaction times for each condition and each group. Mean reaction times and standard deviations are summarized in Table 3. We found a main effect of *Condition* [*F*_(2, 162)_ = 150.17, *p* < 0.001, η*p*^2^ = 0.65], a main effect of *Group* [*F*_(3, 81)_ = 18.75, *p* < 0.001, η*p*^2^ = 0.41], as well as an interaction effect of *Group* × *Condition, F*_(6, 162)_ = 5.31, *p* < 0.001, η*p*^2^ = 0.16. There was no interaction with the factor *Phoneme*, all *Fs* ≤ 0.39, n.s.

**Table 3:** Mean (*M*) and standard deviations (*SD*) for reaction times in ms, for each training group and control group (PHON, phonemic-only group; PHORT, phonemic-orthographic group; CONTROL, control group; ADULT, adult control group) and for each condition (Identity, Variation, Control).

	**Group**			
**Condition**	**PHON *M* (*SD*)**	**PHORT *M* (*SD*)**	**CONTROL *M* (*SD*)**	**ADULT *M* (*SD*)**
Identity	1032.00 (139.33)	1032.40 (139.84)	987.04 (94.09)	801.34 (115.25)
Variation	1078.53 (129.35)	1059.17 (154.95)	1006.14 (100.00)	818.53 (115.63)
Control	1159.08 (146.50)	1141.41 (148.71)	1083.37 (119.21)	846.34 (119.46)

Separate, consecutive *t*-tests in each group revealed a gradual response time pattern across trained as well as untrained phonemes. Participants responded fastest in the Identity condition, intermediate in the Variation condition and slowest in the Control condition. All conditions differed significantly from each other for all groups (PHON: all *ts*_(23)_ ≥ 4.95, *p* ≤ 0.0001, *d* ≥ 0.96; PHORT: all *ts*_(21)_ ≥ 2.87, *p* ≤ 0.009, *d* ≥ 0.67; CONTROL: all *ts*_(20)_ ≥ 2.49, *p* ≤ 0.021; *d* ≥ 0.54; ADULT: all *ts*_(17)_ ≥ 2.94, *p* ≤ 0.009, *d* ≥ 0.60). While the children groups depicted similar overall response times, adult participants responded faster across all conditions (*M* = 822.07 ms, *SD* = 115.46 ms) compared to children from the phonemic-orthographic group (*M* = 1077.66, *SD* = 144.55 ms, *t*_(37.22)_ = 6.21, *p* < 0.0001), as well as compared to the phonemic only group (*M* = 1089.87 ms, *SD* = 134.76 ms, *t*_(39.22)_ = 6.92, *p* < 0.0001) and the children control group (*M* = 1025.52 ms, *SD* = 100.02 ms, *t*_(33.96)_ = 5.83, *p* < 0.0001).

Overall, children made only few errors in word trials, *M* = 4.92%, *SD* = 3.61%, *Range* = 1.01–19.33% (Adults: *M* = 0.47%, *SD* = 0.98%, *Range* = 0.00–4.05%). Error analysis revealed a significant main effect of *Condition* [*F*_(2, 162)_ = 4.09, *p* = 0.019, η*p*^2^ = 0.05] and an interaction effect of *Group* × *Condition, F*_(6, 162)_ = 2.21, *p* = 0.045, η*p*^2^ = 0.08. Children of the phonemic-orthographic group made overall a less amount of total mistakes in the Identity (*M* = 1.38, *SD* = 0.96) and Variation (*M* = 1.27, *SD* = 0.85) condition compared to the Control condition (*M* = 2.02, *SD* = 1.45), both *ts*_(21)_ ≥ 2.41, *p* ≤ 0.02, *d* ≥ 0.09. There was no difference between the Identity and the Variation condition, *t*_(21)_ = 0.48, n.s. In the other groups, there were no significant differences between any of the conditions [PHON: all *ts*_(23)_ ≤ 1.7, n.s.; CONTROL: all *t*_(20)_ ≤ 1.90, n.s.; ADULT: all *t*_(17)_ ≤ 1.14, n.s.].

### Event-Related Potentials

#### 100–300 ms

The ANOVA revealed significant interactions of *Condition* × *Region* [*F*_(2, 162)_ = 9.98, *p* < 0.001, η*p*^2^ = 0.11], of *Group* × *Condition* × *Phoneme* [*F*_(6, 162)_ = 2.39, *p* = 0.031, η*p*^2^ = 0.08], and of *Group* × *Condition* × *Region* × *Hemisphere, F*_(6, 162)_ = 2.47, *p* = 0.026, η*p*^2^ = 0.08. According to the quadruple interaction, we analyzed each group separately.

For the phonemic only group, the ANOVA revealed significant interactions of *Condition* × *Region* [*F*_(2, 46)_ = 4.27, *p* = 0.029, η*p*^2^ = 0.16], and of *Condition* × *Hemisphere, F*_(2, 46)_ = 4.04, *p* = 0.024, η*p*^2^ = 0.15. Over anterior regions, amplitudes in the Identity condition were more negative than amplitudes in the Variation and in the Control condition, all *ts*_(23)_ ≥ 3.05, *p* ≤ 0.005, *d* ≥ 0.71. There was no significant difference in amplitudes between the Variation and the Control condition, *t*_(23)_ = 1.39, n.s. There were no significant differences between the amplitudes over posterior regions, all *ts*_(23)_ ≤ 1.40, n.s. Over the left hemisphere, there was no difference in amplitudes between the Identity and the Variation condition, *t*_(23)_ = 1.01, n.s. However, amplitudes in the Identity and in the Variation condition were both more negative than amplitudes in the Control condition, both *ts*_(23)_ ≥ 2.67, *p* ≤ 0.01, *d* ≥ 0.53. There were no significant differences between amplitudes over the right hemisphere, all *ts*_(23)_ ≤ 0.22, n.s.

For the phonemic-orthographic group, the ANOVA revealed an interaction effect of *Condition* × *Region, F*_(2, 42)_ = 6.94, *p* = 0.002, *np*^2^ =0.25. Similar to the phonemic only group, amplitudes over anterior regions were more negative in the Identity condition than amplitudes in the Variation condition and amplitudes in the Control condition, both *ts*_(21)_ ≥ 2.48, *p* ≤ 0.02, *d* ≥ 0.49. There was no difference between amplitudes in the Variation and Control condition, *t*_(21)_ = 1.76, n.s. A similar pattern emerged over posterior regions: Amplitudes in the Identity condition were more negative compared to amplitudes in the Variation and in the Control condition, both *ts*_(21)_ ≥ 2.08, *p* ≤ 0.04, *d* ≥ 0.41. There was no difference between amplitudes in the Variation and Control condition, *t*_(21)_ = 0.78, n.s.

**Figure 2:**
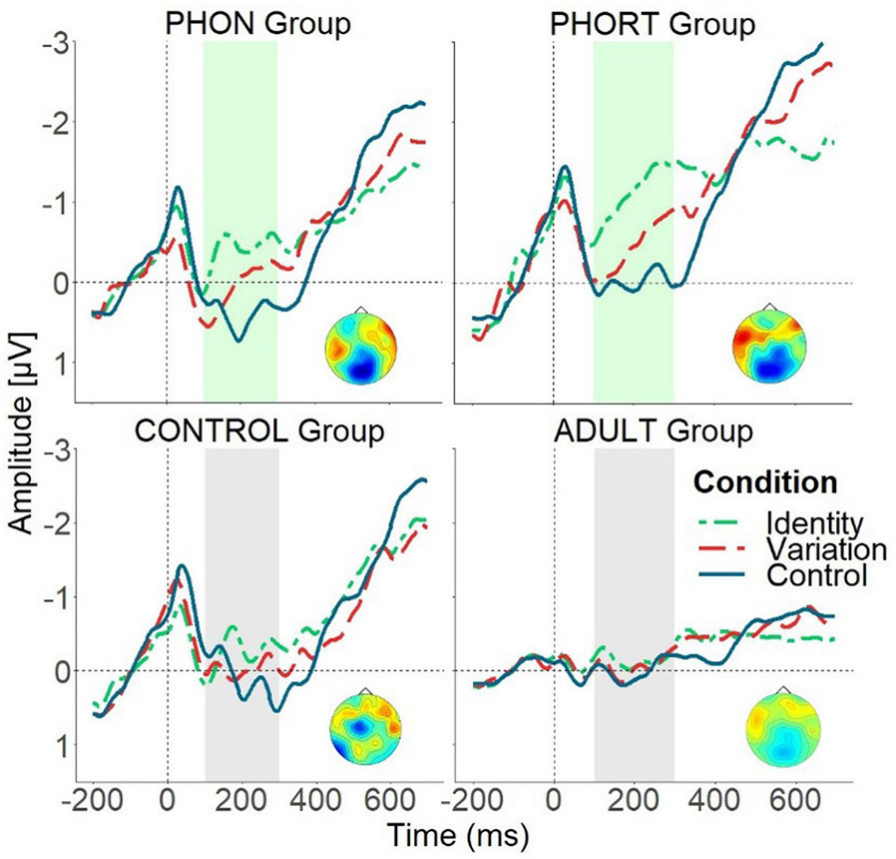
Mean ERP effects over both anterior regions for all four groups separately, illustrating significant ERP differences between groups (see interaction effects including the factor GROUP in the time window between 100 and 300 ms after target word onset). Upper panels: the phonemic-only group (PHON, left) and the phonemic-orthographic training group (PHORT, right). Lower panels: control training group (CONTROL, left) and adult control group (ADULT, right). Identity condition (green, short-dashed line), Variation condition (red, long-dashed line) and Control condition (blue, solid line). The light gray bar marks the analysis area for time window 100–300 ms in which significant group differences were found. The depicted topographical voltage maps indicate significant difference waves for the Variation condition minus Identity condition for the three training groups.

For the children control and the adult group, the ANOVA did not reveal any significant interactions with *Condition*, all *Fs* ≤ 1.51, n.s. Figure 2 illustrates the ERP effects for this time window.

#### 300–400 ms

The ANOVA revealed an interaction of *Condition* × *Phoneme* [*F*_(2, 162)_ = 4.35, *p* = 0.014, η*p*^2^ = 0.05], an interaction of *Condition* × *Region* [*F*_(2, 162)_ = 8.51, *p* < 0.001, η*p*^2^ = 0.10] and a triple interaction of *Condition* × *Region* × *Hemisphere, F*_(2, 162)_ = 5.13, *p* = 0.007, η*p*^2^ = 0.06.

**Figure 3:**
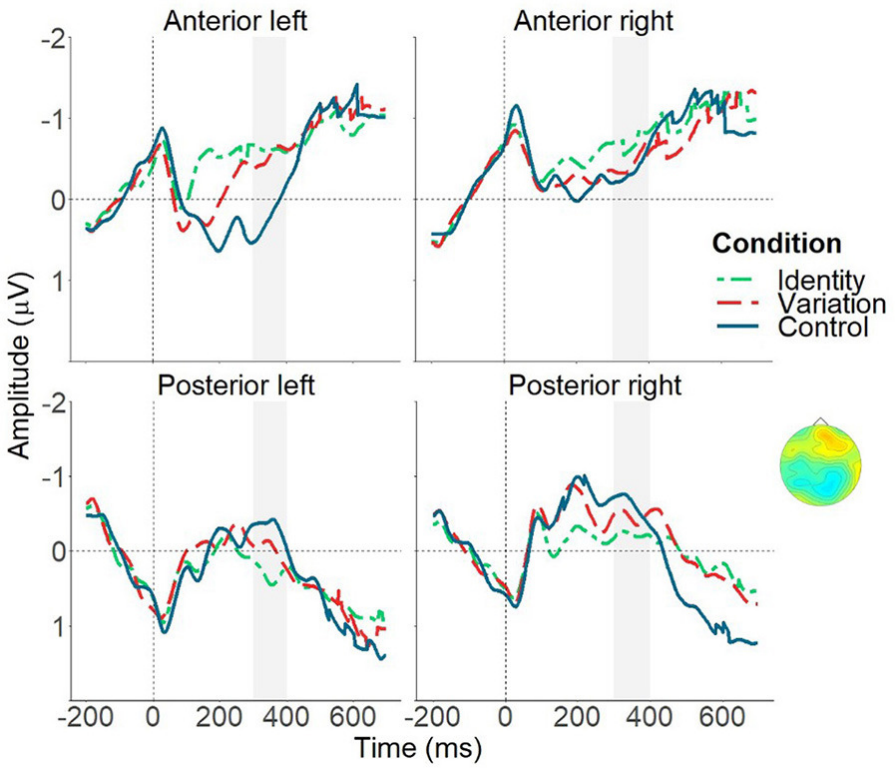
Mean ERP effects over anterior and posterior regions averaged across all four groups (three training groups and adults), illustrating comparable ERP differences for all groups (see interaction effects including condition, but not group in the time window between 300 and 400 ms after target word onset). Identity condition (green, short-dashed line), Variation condition (red, long-dashed line) and Control condition (blue, solid line). The light gray bar marks the analysis area for time window 300–400 ms. The depicted topographical voltage maps indicate significant difference waves for the Variation condition minus Identity condition for the three training groups and adults combined.

Over anterior left regions, amplitudes in the Identity condition did not differ from amplitudes in the Variation condition, *t*_(84)_ = 0.63, n.s. Amplitudes of both conditions were more negative than amplitudes of the Control condition, both *ts*_(84)_ ≥ 3.92, *p* ≤ 0.0001, *d* ≥ 0.45. Amplitudes over the anterior right, posterior left and posterior right regions were more negative (positive) in the Identity condition compared to the Control condition, all *ts*_(84)_ ≥ 1.99, *p* ≤ 0.04, *d* ≥ 0.22. There were no differences of amplitudes between the Identity and Variation condition, or Variation and Control condition in these regions, all *ts*_(84)_ ≤ 1.89, n.s. Figure 3 illustrates the ERP effects over anterior and posterior regions across all groups.

In sum, between 100 and 300 ms after target word onset, differences in amplitudes arose between groups. Both phonemic groups, but not the training control or adult group, showed differences between the Identity condition and the Variation condition over anterior regions. In the combined training group, a similar pattern of amplitude differences emerged over posterior regions. In a later time window (300–400 ms), all children as well as adult participants, exhibited a robust priming difference between the Identity and the Control condition, but the critical conditions did not differ (Identity vs. Variation). We neither found main effects of the factor *Phoneme*, indicating different ERP results for trained vs. untrained phonemes, nor any interactions including this factor.

## Discussion

In this intervention study, we investigated whether phonemic awareness alone (*implicit phonological account*) or in combination with grapheme knowledge (*implicit orthographic-phonological account*) shapes implicit phonological processing in preliterate children, or whether phonemic awareness rather leads to more elaborated strategic processing beyond obligatory processing levels of speech recognition (*explicit phonological account*). For this purpose, we provided 10 weeks of daily training sessions to three groups of preliterate children: (i) one group received a training on phonemic awareness only (phonemic group), (ii) another group received a combined training on phonemic awareness and on some letters (phonemic-orthographic group), and (iii) a third group received a non-linguistic training on finger-number associations (control group). Thereafter, we tested different aspects of speech processing in these differently trained children.

Results of the explicit measurements showed improvement of overall phonological awareness in both phonemic training groups in comparison to the control group, but no specific training effects on phonemic awareness or letter knowledge. Reaction times indicated differentiated processing of voicing variations in all training groups as well as in the control adult group. However, at early stages of phonological processing, children of both phonemic training groups appeared to process phonemic variations in more detail, whereas children of the control group and adults showed no differentiated processing of voicing mismatches. In the following discussion, we at first will take a closer look at the ERP results of the control training and the control adult group.

### Can Previous Findings on Phonological Sensitivity to Phonemic Mismatch be Generalized to a Different Phonemic Feature?

The present data of young adults only partly replicated previous results of two auditory word onset priming studies investigating variation in place of articulation (Friedrich et al., 2009; Schild et al., 2012). Similar to both previous studies, adults of the present study showed a graded pattern in their reaction times. They responded fastest in the Identity condition, slower in the Variation condition and slowest in the unrelated Control condition. This suggests that adults noticed prime-target variation in voicing and that this partial mismatch interfered with the yes-decision required in the lexical decision response. However, adults did not show ERP differences between the Identity condition and the Variation condition. This result was not expected on the basis of both previous studies on place variation, which had shown a pattern of graded ERP in parallel to a graded pattern in reaction times. Apparently, it makes a difference whether phonemic variation occurs in place of variation (former studies) or voicing (present study).

The ERP results of the present study in young adults suggest that phonemic variation in voicing might not be considered at early stages of processing including stages that we formerly considered to reflect lexical access. Variations in place of articulation modulated speech processing between 300 and 400 ms after stimulus onset (e.g., Friedrich et al., 2009); whereas in the present study variations in voicing only produced a priming effect (differentiating the Identity and the Control condition) in a similar time window without a graded modulation of variations of voicing. Thus, it seems that the priming *per se* worked, but in the present study variations of voicing did not occur—as it was intended in the lexical access process mirrored in the P350 that we found in the former study with adults (Friedrich et al., 2009; Schild et al., 2012). Whether this different use of both articulatory features in lexical access is driven either by their special acoustic cues (e.g., amongst other things varying voice onset times in voicing contrasts and rapid formant transitions varying for different consonant-vowel pairing in place contrasts) or by their abstract linguistic representations is difficult to disentangle, but interesting to address in future research.

Again, ERP and reaction time data recoded in word onset priming appear to dissociate functionally different aspects of speech processing and decision making. While ERP reflected privileged processing of partially mismatching targets (compared to unrelated targets) across several studies with adults, decision latencies for partially mismatching targets were not facilitated in some studies (Friedrich et al., 2008; Schild et al., 2014a; Kóbor et al., 2018) or even inhibited in one study (Friedrich et al., 2013). That is, in former studies, processing reflected in the ERP was more tolerant to subtle phonological variation than processing reflected in lexical decision latencies. The present results for young adults fit into this line of research in that aspects of input-mediated lexical access (which seem to be reflected in the ERP; e.g., Friedrich, 2005; Friedrich et al., 2008, 2009, 2013; Bien et al., 2014; Schild et al., 2014b; Kóbor et al., 2018; Schild and Friedrich, 2018).

Results obtained for children receiving a non-linguistic training were comparable to that of young adults. While we found a graded activation pattern in the reaction times (indicating sensitivity to phonemic mismatch), children who participated in a non-linguistic training did not seem to consider voicing differences for input-mediated lexical access, as shown in similar ERP amplitudes elicited in the Identity condition and the Variation condition in the time window between 300 and 400 ms after stimulus onset. We might conclude that adults and preliterate children process the feature voicing similarly: They only consider voicing variations at a late, strategic stage of processing, but not necessarily at an early processing level. Note, that both groups showed signs of priming *per se* between 300 and 400 ms indicating that the ERPs reflect part of the priming. Together the findings for both control groups can be considered as some evidence for an *explicit phonological account* to lexical decision latencies (e.g., Cutler et al., 2010; Cutler and Davis, 2012; Mitterer and Reinisch, 2015). Similar to young adults, children who were not phonemically trained seemed to consider voicing mismatches at an explicit decisional level, but they did not seem to use it at an early processing stage.

Before we interpret the results of both phonemic trainings, we need to reflect on how efficient both linguistic trainings were.

### How Did the Training Affect the Performance on the Explicit Measurements?

Evidencing that the phonemic training worked, both phonemic training groups improved in phonological awareness (including phonemic awareness subtests) compared to the non-linguistic training group. Former studies applying phonological trainings over 6 to 8 months had already reported sustainable effects on the performance in explicit metalinguistic tasks in preliterate children, in comparison to a group without training (Lundberg et al., 1988; Schneider et al., 1997). Note, that these former studies included also training of larger speech segments (e.g., syllables) in their intervention program, resulting in an overall longer duration of the training itself; the duration of training on actual phonemic awareness was roughly comparable to that of our study. Compared to these studies, we observed comparable effects sizes of the training on phonological awareness measurements. However, in general, all three training groups gained expertise in phonemic awareness from pre- to post-training testing; specifically all children improved in detecting single onset phonemes from pre-training to post-training, including the sounds that were crucial in the experiment (/g/, /k/, /b/, /p/). This implies developing phonemic awareness in middle childhood. Presumably triggered by a general interest in school-related activities (such as interest in learning letters, in playing word games or in reading), preliterate children might become aware of phonological units like rhymes, syllables or phonemes even without specific training. Furthermore, given that the children were tested with the same material at pre- and post-test, it cannot fully be ruled out that re-testing effects might have at least partially contributed to the overall time effect in the phonemic awareness and the letter knowledge test. The children also were tested with the same version of the TEPHOBE test for phonological awareness at post-testing. Here, the interaction effect suggests that re-testing might not solely drive the effect.

Somewhat unexpectedly, the combined phonemic-orthographic training did not specifically improve in explicit letter knowledge. Again, all groups improved from pre- to post-training testing. However, in contrast to particularly enhanced phonological awareness in both phonemic training groups, children did not gain particularly enhanced letter knowledge from the phonemic-orthographic training. It appears that, regarding grapheme knowledge, a 10-week training is not as effective as longer interventions are. Former studies applying phonological trainings with a focus on the correspondence between letters and speech sounds reported efficient letter learning together with sustainable effects on the performance in explicit metalinguistic tasks (Bus and van Ijzendoorn, 1999; Schneider et al., 2000). In the present study, it might have been the case that letter knowledge was not sufficiently established by the short exercises of the phonemic-orthographic training. Alternatively, already prior to the training children showed a substantial level of letter knowledge which might have reduced eventual training effects due to ceiling effects (see Table 2). Finally, as we found that the phonemic-orthographic group outperformed the control group in the amount of capital letters children know (over both pre- and post-tests), we could at least speculate that differences especially between these two groups could be related to the amount of orthographic knowledge.

Nonetheless, regarding our research questions and the discussion of the present ERP and behavioral results, we should keep in mind that results of explicit measures indicate that the training itself did improve phonemic knowledge, but failed to establish robust letter-speech sound associations *above* the level that children of this age typically reach.

### Does a Training of Phonemic Awareness Alone and/or Combined Training of Phonemic Awareness and Letter Knowledge Lead to More Detailed Speech Processing in Preliterate Children?

In contrast to ERP obtained from adults and from children receiving a non-linguistic training, ERPs obtained from children of both phonemic training groups differed for the Identity condition and the Variation condition. Yet, similar to adults and untrained children, we did not replicate ERP differences in the P350 time window, ranging from 300 to 400 ms and linking to lexical access (as in both former word onset priming studies with adults). ERP differences between the Identity and the Variation condition found in the phonemic training groups emerged already between 100 and 300 ms after target word onset. This time window is associated with the N100/P200 and its temporally distributed counterpart—the T-complex, both linking to early auditory and phonological analysis of the speech signal and to auditory attention mechanisms (Wolpaw and Penry, 1975; Näätänen and Picton, 1987; Connolly, 1993; Poeppel et al., 1997; Diesch and Luce, 2000; O'Rourke and Holcomb, 2002; Sanders et al., 2002). Given that adult proficient readers did not consider these voicing differences for early processing, but children from both phonemic trainings did, this possibly implicates that the phonemic trainings might have enhanced early phonological analysis through enhanced auditory attention, as both of the phonemic training groups had been specifically sensitized for voicing contrast. This, however, implicates that rather strategical mechanisms as increased attentional processes might have affected early priming as found in the phonemically trained children. As the adult listeners in this study did not seem to engage with these differences in voicing when attending to spoken language, we might conclude that instead of changes in implicit processing through phonological restructuring, a rather explicit and possibly temporary attentional mechanism underlies these results.

Do newly learned graphemes add more detail to phonological processing in preliterate children than phonemic awareness alone? Our results of ERP amplitudes rather speak against this hypothesis, as we found comparable ERP evidence for more detailed early speech processing in both groups receiving a phonemic training. However, as we did not find notable training effects in letter knowledge of the combined training group, this result is difficult to interpret. Moreover, due to the discussed ceiling effects of the letter knowledge we cannot exclude the possibility that both training groups already built up grapheme representations, which might have affected the early processing of the words, so the interpretation of the results need to be regarded with caution.

However, although the combined training group outperformed the control group in letter knowledge, the phonemic training group did not differ in letter knowledge neither from the combined training group nor from the control group. This and the fact that ERPs look similar for both trained groups seem to be, in turn, more in line with the interpretation that phonemic knowledge rather than letter knowledge caused the differential ERP effects. Additionally, we also did not find different results for trained phonemes (/g/ and /k/) vs. untrained phonemes (/b/ and /p/), neither for the phonemic nor for the combined phonemic-orthographic training. Prime-target mismatch in trained and untrained phonemes elicited a comparable pattern of behavioral responses and ERPs. Importantly, the modulated phonological processing observed in the present study seems neither restricted to the specifically trained phonemes nor related to newly acquired grapheme representations. This might reflect generally enhanced phonological processing. Alternatively, results might reflect feature-based attenuation resulting from the trained differentiation between phonemes differing only in voicing. This sensitivity to voicing differences might then have generalized to untrained phonemes.

Our present results are not entirely clear with regard to the previously found enhanced sensitivity to phonological detail in reading children compared to pre-readers (Schild et al., 2011). Even if we should consider that the trained children knew some of the letter-sound connections as discussed above, it is still unlikely that their proficiency in letter and sound knowledge equals the reading expertise of the reading children groups tested in the study of Schild et al. (2011). Phonological proficiency acquired via our phonemic trainings might not be comparable to phonological proficiency in beginning readers. In the present training study, we provided a relatively short intervention program, in which children who had very rudimentary overall letter knowledge learnt about a few grapheme-phoneme correspondences. We trained only a few, selected letters. This is clearly different from the children in the study by Schild et al. (2011), who were already reasonably proficient readers (i.e., pre-school children with early reading skills and second graders). Two issues need to be addressed here: First, instructing only a few letters might not have established sufficiently profound letter knowledge in children of the combined phonemic-orthographic training group. Moreover, one might speculate that grapheme knowledge only becomes relevant for lexical access after a more intense training and/or at a more advanced level of literacy acquisition. On the other hand, ERP differences obtained in the present study (N100/P200 effects) did not correspond to ERP differences obtained in the previous study (P350 effect). Given our failure to replicate the graded P350 effects formerly found for variation in place of articulation, it appears that the feature voicing was not optimally chosen for a comparison of the former and the present study. Consequently, we cannot completely rule out the interpretation that the formerly obtained P350 effect was related to orthographic knowledge.

It remains possible that implicit links between speech sounds and graphemes develop even after pre-school age at an advanced level of literacy. Some neurocognitive data on speech-sound spelling interactions support such a long-lasting plasticity. *Mismatch negativity* (MMN) responses in the ERP revealed implicit links between phonemic and orthographic representations in adults: Visual letters enhanced automatic auditory MMN responses (Froyen et al., 2009). However, visually enhanced auditory MMN responses were not obtained in beginning readers and the timing of this effect was somewhat delayed even in 4th graders compared to adults (Froyen et al., 2008). This suggests that automatic grapheme-phoneme connections might develop relatively slowly up to late childhood. In line with this, spelling biases (e.g., Pattamadilok et al., 2007, 2014; Ziegler et al., 2008) develop relatively slowly, with “fully developed” spelling biases emerging no earlier than from 3rd to 4th grade (Ventura et al., 2007, 2008).

For now, our results do not allow to rule out the possibility that grapheme representations affect phonological processing, given that all children had substantial letter knowledge after the trainings. However, there are hints that early enhanced phonological processing might rather be related to attentional mechanisms which seem to be foremost related to the phonemic trainings and increased phonological awareness, as control group children (albeit performing similarly on the letter knowledge test) did not show early priming effects in the ERP analysis. Future research will be needed to answer the questions whether this early enhanced processing is either only short-termed and an immediate modification of the phonological trainings, or whether this effect is long-lasting and may relate to an improved literacy acquisition. If and how restructuring processes are involved as assumed by the *implicit phonological account* (e.g., Harm and Seidenberg, 2004; Taft, 2006; Pattamadilok et al., 2010; Dehaene et al., 2015) is difficult to answer. Given that adult proficient readers do not consider voicing variations neither during lexical access nor in early phonological analysis, listeners might process these variations only at a very early, exercises-intensive level of literacy acquistion.

### Conclusion

Here, we conducted the first training study trying to disentangle the influence of orthographic knowledge and phonological awareness on the plasticity of speech perception during the very beginnings of reading acquisition in the last year of kindergarten. Children receiving a phonemic training and those receiving a phonemic-orthographic training exploited phonemic detail for more aspects of speech processing than children receiving a non-linguistic intervention. This enhanced processing was restricted to early stages of phonological analysis. Our results indicate that enhanced phonological awareness—developing as a precursor function and in connection with reading—might modulate attention mechanisms and phonological analysis in speech recognition in pre-reading children. As our results are only informative for pre-reading children, it will be of interest whether grapheme information might play a stronger role once letter-sound correspondences are more stable.

## Data Availability Statement

The raw data supporting the conclusions of this article will be made available by the authors, without undue reservation.

## Ethics Statement

The studies involving human participants were reviewed and approved by Ethikkommission der Deutschen Gesellschaft für Psychologie. Written informed consent to participate in this study was provided by the participants' legal guardian/next of kin.

## Author Contributions

US conceived the study. AB took primary responsibility for drafting the manuscript and conducted the study and analyzed the data. AB, CF, and US contributed to design of the training and the tasks. CF and US commented on drafts. All authors read and approved the submitted version.

## Conflict of Interest

The authors declare that the research was conducted in the absence of any commercial or financial relationships that could be construed as a potential conflict of interest.
