# Supplementary material for: Phonemic Training Modulates Early Speech Processing in Pre-reading Children
Source: Front Psychol. 2021 Jun 1;12:643147. doi: 10.3389/fpsyg.2021.643147 (PMC8205151; doi:10.3389/fpsyg.2021.643147)
Supplement: Supplementary file 1 [file Table_1.DOCX]

**Supplementary Material**

Table S1.

*List of games used in the phonemic trainings*

| Game name | Example | Times |
| --- | --- | --- |
| **Exercises on Initial Sounds** | Identifying the first sound in the names of each child. | 3 |
| **Sound-Assignments: Story** | Repeating specific sounds mentioned in a story and paying attention how it is formed with the mouth. Example: "Imagine you are playing outside and you find an easter egg (German: Osterei). You say ‘OOH there’s an easter egg (Osterei)!’ Your lips form a circle when you say /o/.” | 14 |
| **Sound-Assignments: Pictures** | Guessing the first sound of an object or animal featured on a picture card. | 6 |
| **I am thinking of …** | Guessing an object or animal that starts with a specific sound. Example: “I am thinking of something starting with /g/. It is transparent and appears at midnight.” (Solution: “Ghost”) | 6 |
| **Loading Ships** | Identifying the initial sound of an object or animal displayed on a card and assign them to a paper ship that features this sound. Example: “All things starting with /g/ belong in the ‘/g/ ship’, all things starting with /k/ in the ‘/k/ ship’.” | 6 |
| **We are looking for…** | Finding words that start or contain a specific sound. | 6 |
| **Pay attention to the first sound** | Listening to a word and identifying the first sound. | 4 |
| **Which word do I mean?** | The trainer segments a word into its phonemes. Children shall identify the word. Example: “Which word do I mean? ‘K-I-W-I’.” | 4 |
| **Game of Dice** | Rolling a dice with objects/animals on the sides. Identifying the first sound of an object or animal that is featured on the dice. | 6 |
| **Picture Tower** | Sorting cards to a “tower” depending on the initial sound of the featured object or animal. | 6 |
| **Picture Cards** | Picking a card and identifying the first sound of the featured object or animal | 6 |
| **Domino** | Connecting domino cards that feature objects or animals starting with the same sound. | 10 |
| **Lottery** | Each child receives a picture card featuring an object or animal with a different sound and collects other cards that also start with that sound. | 7 |
| **The Ball** | The trainer tosses a ball to one child and tells the child a word segmented into its phonemes. The child shall guess the word. Example: “What word do you hear? ‘K-U-SS’ (*kiss*)”. | 5 |
| **Phoneme segmentation** | Segmenting words into their single phonemes. Example: “Which sounds do you hear in the word ‘Oma’? (*grandma*)”. | 5 |

*Note.* List of games used in the phonemic trainings, including examples and number of runs they had in the program (times). In the exercises, we only used words starting with /a/, /e/, /i/, /o/, /g/ and /k/. In the combined training group, we additionally inserted a letter card to each sound. We took care that the instructors did not use any of the words from the experiment as examples in the training. Games are listed in chronologically order of appearance in the intervention.

Table S2.

*List of stimuli*

| Target words | Pseudowords | Target words | Pseudowords |
| --- | --- | --- | --- |
| Gei-er (vulture) | Geine | Pap-pe (cardboard) | Papke |
| Gei-ge (violin) | Geise | Pul-ver (powder) | Pulbel |
| Git-ter (grid) | Gitsche | Pum-pe (pump) | Pumle |
| Gloc-ke (bell) | Glocpe | Pud-ding (pudding) | Pudhe |
| Gra-ben (trench) | Grany | Pup-pe (doll) | Pupte |
| Gren-ze (border) | Grenhe | Pic-kel (pimple) | Picsche |
| Gru-be (pit) | Gruza | Po-ny (pony) | Poben |
| Grup-pe (group) | Grupzle | Piz-za (pizza) | Pizbe |
| Guer-tel (belt) | Guerbe | Pan-ne (breakdown) | Panze |
| Gum-mi (rubber) | Gumse | Peit-sche (whip) | Peitter |
| Gur-ke (cucumber) | Gurbon | Pom-mes (fries) | Pombel |
| Kaff-ee (coffee) | Kaffnen | Bri-lle (glasses) | Brissen |
| Ka-ter (male cat) | Kaffel | Bie-ne (bee) | Bieer |
| Kat-ze (cat) | Katne | Brun-nen (fountain) | Brunnee |
| Ker-ze (candle) | Kertel | Bon-bon (candy) | Bonke |
| Ket-te (chain) | Ketzel | Bru-der (brother) | Bruchen |
| Keu-le (mace) | Keusen | Bam-bus (bamboo) | Bamsche |
| Ki-no (cinema) | Kite | Ba-by (baby) | Bave |
| Kir-che (church) | Kirber | But-ter (butter) | Butche |
| Kir-sche (cherry) | Kirbus | Bue-gel (stirrup) | Buede |
| Kis-sen (pillow) | Kisle | Bruec-ke (bridge) | Bruecfer |
| Kis-te (box) | Kiskel | Brem-se (break) | Bremken |
| Kno-chen (bone) | Knoder | Buer-ste (brush) | Buerno |
| Kno-ten (knot) | Knore | Brau-se (shower) | Braunig |
| Koe-nig (king) | Koese | Bom-be (bomb) | Bomtel |
| Kof-fer (trunk) | Kofke | Bir-ne (pear) | Birgel |
| Krae-he (crow) | Kraeding | Bue-hne (stage) | Buehmel |
| Kraeu-ter (herbage) | Kraeude | Blu-me (flower) | Bluchen |
| Kral-le (claw) | Kralpe | Blue-te (blossom) | Bluene |
| Krei-de (chalk) | Kreigel | Buef-fel (buffalo) | Buefter |
| Kroe-te (toad) | Kroepe | Be-sen (broom) | Bele |
| Kro-ne (crown) | Krote | Blu-se (blouse) | Bluge |
| Krue-mel (crumbs) | Kruehne | Bla-se (bubble) | Blami |
| Kue-che (kitchen) | Kueter | Bi-ber (beaver) | Biche |
| Kue-ken (chicken) | Kuekse | Bee-re (berry) | Beerten |
| Kur-ve (curve) | Kurby | Beu-tel (bag) | Beuze |
| Kut-sche (carriage) | Kutkel | Bre-zel (pretzel) | Brete |

*Note*. Full list of stimuli (target words and pseudowords) that were used in the priming experiment.

Table S3.

*Reaction times and error ANOVA analysis for the three training groups without the adult control group*

|  | Test statistics | | |
| --- | --- | --- | --- |
| Factor | *F* | *p* | *ηp^2^* |
| **Reaction times** | | | |
| G | 1.55 | .21 | .05 |
| C | 141.31 | **< .001** | .69 |
| P | 1.06 | .30 | .02 |
| G x C | 1.15 | .33 | .03 |
| G x P | 1.42 | .24 | .04 |
| C x P | .31 | .73 | <.01 |
| G x C x P | .39 | .81 | .01 |
|  |  |  |  |
| **Error analysis** |  |  |  |
| G | 2.63 | .08 | .08 |
| C | 4.28 | **.01** | .06 |
| P | 18.55 | **< .001** | .22 |
| G x C | 2.31 | .06 | .07 |
| G x P | .31 | .81 | .01 |
| C x P | 3.00 | **.05** | .04 |
| G x C x P | 2.29 | .06 | .07 |

*Note.* Factor abbreviations are as following: C = *Condition*, G = *Group*, P = *Phoneme*, R = *Region*, H = *Hemisphere*. Significant results are marked in bold.

Table S4.

*ERP ANOVA analysis for both time windows (100 - 300 ms and 300 - 400 ms) for the three training groups without the adult control group*

|  | Test statistics | | |
| --- | --- | --- | --- |
| Factor | *F* | *p* | *ηp^2^* |
| **100 - 300 ms** | | | |
| C | 10.05 | **< .001** | .14 |
| G x C | 1.90 | .11 | .06 |
| C x P | 3.41 | **.03** | .05 |
| C x R | 10.53 | **< .001** | .14 |
| C x H | 2.86 | .06 | .04 |
| G x C x P | 2.57 | **.04** | .07 |
| G x C x R | .71 | .58 | .02 |
| G x C x H | .67 | .61 | .02 |
| C x P x R | .51 | .60 | .01 |
| C x P x H | 1.88 | .15 | .03 |
| C x R x H | .26 | .77 | <.01 |
| G x C x P x R | 1.97 | .10 | .06 |
| G x C x P x H | 1.87 | .12 | .06 |
| G x C x R x H | 2.88 | **.02** | .08 |
| C x P x R x H | .02 | .97 | <.01 |
| G x C x P x R x H | 1.21 | .30 | .04 |
|  |  |  |  |
| **300 - 400 ms** |  |  |  |
| C | 4.00 | **.02** | .06 |
| G x C | 1.64 | .16 | .05 |
| C x P | 4.52 | **.01** | .07 |
| C x R | 7.39 | **.001** | .10 |
| C x H | .58 | .56 | .01 |
| G x C x P | 2.24 | .06 | .07 |
| G x C x R | .10 | .98 | <.01 |
| G x C x H | 1.07 | .37 | .03 |
| C x P x R | .43 | .65 | .01 |
| C x P x H | 1.19 | .30 | .02 |
| C x R x H | 5.46 | **.005** | .08 |
| G x C x P x R | 1.08 | .36 | .03 |
| G x C x P x H | .97 | .42 | .03 |
| G x C x R x H | 1.33 | .26 | .04 |
| C x P x R x H | .72 | .48 | .01 |
| G x C x P x R x H | .76 | .55 | .02 |

*Note*. For a better overview, only main effects and interactions with the factor CONDITION are displayed. Factor abbreviations are as following: C = *Condition*, G = *Group*, P = *Phoneme*, R = *Region*, H = *Hemisphere*. Significant results are marked in bold.
